# *Escherichia coli* type I toxin TisB exclusively controls proton depolarization following antibiotic induced DNA damage

**DOI:** 10.1038/s41598-025-96136-x

**Published:** 2025-04-14

**Authors:** Tekle Airgecho Lobie, Charlotte Solum Krog, Kirsten Skarstad, Magnar Bjørås, James Alexander Booth

**Affiliations:** 1 Department of Microbiology, University of Oslo, and Oslo University Hospital, Rikshospitalet, Oslo, Norway; 2 Department of Clinical and Molecular Medicine, Norwegian University of Science and Technology, Trondheim, Norway; 3 Centre for Embryology and Healthy Development, University of Oslo, 0373 Oslo, Norway

**Keywords:** TisB, Membrane depolarization, Type I toxin antitoxin systems, Antibiotics, DNA damage, *E. coli*, Microbiology, DNA damage and repair

## Abstract

Bacterial toxin-antitoxin (TA) systems are genetic loci where the antitoxin gene product helps to control the expression or activity of the toxin gene product. Type I TA systems typically produce hydrophobic peptides that often localize to the inner membrane of bacteria. These amphipathic peptides can then potentially affect ion flows across the inner membrane. Here, we show that several type I toxins from *Escherichia coli* can affect depolarization, whereas *tisB* exclusively controls the depolarization of the proton gradient. *tisB* has been linked to persister cell formation following treatment with the antibiotic ciprofloxacin and *tisB-istR* has been implicated in the control of proton depolarization following treatment with ofloxacin. These results suggest that *tisB* could initiate the formation of persister cells by fully dissipating the proton gradient and that most of the electrical gradient greatly limiting ATP production following antibiotic-induced DNA damage.

## Introduction

Bacterial genomes contain many enigmatic genetic loci known as toxin-antitoxin (TA) systems that have only been annotated in the recent past^1^. Type I TA systems produce a peptide that is often toxic upon overexpression and an RNA antitoxin that removes the translationally active transcript of the peptide by RNase III cleavage^2^. Many type I TA systems produce hydrophobic toxins that localize to the inner membrane. Examples of the *E. coli* strains studied here include *dinQ-agrB*^3,4^, *ldrD-rdlD*^5,6^, *shoB-ohsC* and the *ibsA-E* systems^7^. One of the best studied examples in *E. coli* is the DNA damage response sensitive *tisB-istR* system^8^. Overexpression of *tisB* resulted in the localization of the peptide in the inner membrane where it led to the destabilization of 23S and 16S rRNA, inhibition of transcription, translation and replication, to reductions in intracellular concentrations of ATP and ultimately bacterial death^9^. Overexpression of *tisB* was also shown to have inhibitory effects on the DNA damage response by increasing the levels of LexA which resulted in reduced induction of prophage λ, reduced filamentation and reduced mutagenesis^10^. DNA damage induced by ciprofloxacin triggers a 1,000-fold increase in *tisB* promoter activity, as measured by a plasmid based *gfp* reporter system. Subsequently, *tisB* has been implicated in the formation of antibiotic-tolerant persister cells^11^. *tisB* was hypothesized to affect the proton motive force (PMF) as an explanation for the decrease in ATP concentration. Experimental results have shown that TisB can form anion-conductive pores or dimers in in vitro lipid bilayers^12–14^.

Recently, *tisB* was shown to be responsible for multiple downstream effects of ofloxacin treatment. *tisB* mutants fail to depolarize either their electrical or proton gradients or undergo cytoplasmic condensation^15^. Previously, we provided evidence of the importance of equilibration of the pH gradient following antibiotic induced DNA damage and how pH equilibration affects morphology, mutagenesis and membrane stability^16^. Using a similar methodology, we investigated which type I TA systems were responsible for pH equilibration and electrical depolarization following exposure to the DNA damaging antibiotic nalidixic acid. Here, we studied membrane potential dynamics under acidic conditions that mirror environments *E. coli* naturally encounters, such as the acidic pH of the duodenum. These conditions allowed us to investigate the physiologically relevant integration of pH sensing with DNA damage responses. Our study investigated type I toxins, which are small amphipathic hydrophobic peptides that may impact membrane homeostasis and ion gradients. While we focused primarily on directly SOS-regulated systems like *tisB*-*istR* and *dinQ*-*agrB*, we broadened our scope to include related type I systems due to the expansive nature of the DNA damage response network and the documented regulatory cross-talk between these systems^10,17^. Here, we show that several type I TA systems can influence electrical depolarization following nalidixic acid treatment. However, as proposed, *tisB* appears to be solely responsible for equilibration of the proton gradient. This causes *tisB* mutants to have an overactive SOS response, especially in the later stages, leading to excess *umuDC* and elevated mutagenesis. Finally, we suggest that *tisB* initiates persister formation following a hierarchical process induced by DNA damage. Nalidixic acid inhibits topoisomerases resulting in replication fork collapse and the production of double strand breaks^18^. These breaks are recognized by RecBCD and processed to form ssDNA and in the presence of RecA nucleoprotein filaments are formed^19^. These filaments then facilitate the autoproteolytic degradation of LexA^20^. Falling concentrations of LexA result in the asymmetric derepression of DNA damage inducible genes, including *tisB*, resulting in a large increase in *tisB* transcription and ultimately the expression of the TisB toxin^11^. We now believe that TisB is exclusively responsible for the depolarization of the proton gradient and contributes substantially to the depolarization of the electrical gradient. Our, and others, hypothesis is that this reduces the metabolic activity in bacteria which can results in a persister state and increased tolerance towards antibiotics that require active growth for efficacy.

## Materials and methods

### Strains and microbial techniques

The bacterial strains and plasmids used are listed in Table S1, and the oligonucleotides used for recombineering are listed in Table S2. The transfer of genotypes of interest was carried out by P1 transduction^21^. The transformation of plasmids was carried out via electroporation using electrocompetent cells generated using a glycerol/mannitol density step centrifugation^22^. Genetic antibiotic selection markers flanked by FRT sites were removed using the FLP recombinase containing plasmid pCP20^23^. Antibiotics were used at the following concentrations, kanamycin 50 µg/ml (plasmid selection) 30 µg/ml (genomic selection), ampicillin 100 µg/ml (plasmid selection), rifampicin 100 µg/ml and nalidixic acid 100 µg/ml (optimum concentration for lethality)^24^. LB broth (10 g of tryptone, 5 g of yeast extract, and 10 g of NaCl adjusted to 1 l with MQ H_2_O and autoclaved) was used for none pH adjusted experiments together with agar (1.5%) if for solid media. LBK (10 g of tryptone, 5 g of yeast extract, 7.45 g KCl, adjusted to 1 l with MQ H_2_O and autoclaved) was used to limit the sodium ion concentration which inhibits growth at high pH^25,26^. The pH values of the LBK, M9 minimal media and PBS solutions were adjusted (100 mM) with Good’s sulfonate buffers, 2-(N-morpholino)ethanesulfonic acid hydrate (MES) was used at pH 5.2, 5.5 and 6; 3-(N-morpholino)propanesulfonic acid (MOPS) for pH 7; and N-[tris(hydroxymethyl)methyl]-3-aminopropanesulfonic acid (TAPS) was used at pH 8. Adjustments were carried out with KOH (5 M) and the solutions were sterilised by filtration (0.2 µm).

### Strain construction

Recombineering was carried out in BW25113 cells electroporated with pSIM6^27^. The oligonucleotides (Table S2) required were designed to remove the gene and its promoter while leaving terminator structures intact to minimise transcriptional effects on adjacent genes.

### Flow cytometry – DiBAC_4_(3) staining

The bacterial strains were grown (37 °C, 200 rpm) in pH-adjusted media (LBK, 3 ml in a 15 ml tube) to an OD_600_ of 0.6 or the indicated OD_600_ (Nanodrop, ND-1000). At the appropriate OD_600_ nalidixic acid was added. The samples were diluted in PBS (100 µl) buffered to the same pH as the incubation media and supplemented with DiBAC_4_(3) (2 µM) after five hours of incubation (37 °C, 200 rpm). A constant DiBAC_4_(3)-to-bacteria ratio was maintained, so typically, 2 µl of sample was added from an OD_600_ = 0.6 culture^28^. The fluorescence signals from 10,000 bacteria/sample were measured on an AccuriC6 flow cytometer in FLA-1, and FSC-A values were simultaneously recorded to determine the fluorescence density. A typical gating of the bacteria is shown in Figure S1.

### Flow cytometry – SOS gene promoter GFP fusion experiments

Bacterial strains containing either plexA-gfp or pumuDC-gfp^29^ were grown (37 °C, 200 rpm) in media (LBK, MES-KOH pH 5.7, 3 ml in a 30 ml tube) to OD_600_ = 0.6 (NanoDrop, ND-1000). The first sample was taken before the addition of nalidixic acid and designated as the zero minutes time point. The samples (2 µl) were diluted in PBS (100 µl) buffered to the experimental pH, typically pH 5.7, and pH 8 together with the membrane permeable weak acid sodium benzoate (60 mM), which acts as a proton ionophore, anchoring the intracellular pH at pH 8. The fluorescent signal of Gfp is maximized at pH 8, increasing the sensitivity of the assay^16^. The fluorescence signals from 10,000 bacteria/sample were measured on an AccuriC6 flow cytometer in FLA-1, and FSC-A values were simultaneously recorded to determine the fluorescence density^30,31^. The activity of the *lexA* or *umuDC* promoter was quantified by calculating the fluorescence density at pH 8, which also corresponds to the amount of Gfp in the bacteria at pH 8. pH depolarization was determined by calculating the difference in fluorescence density measurements between those at pH 8 and at the intracellular pH and subsequently correcting for the amount of Gfp.

### Microscopy – filamentation

BW25113 and *tisB* and *istR* mutant bacterial strains were grown (37 °C, 200 rpm) in media (LBK, MES-KOH pH 5.7, 3 ml in a 30 ml tube) to an OD_600_ of 0.6 (Nanodrop, ND-1000). The first sample was taken before the addition of nalidixic acid and designated the zero minutes time point. Aliquots of the bacterial culture were fixed at a 1:1 ratio with cold 94% ethanol at the specified time points. Cover slides were prepared by coating them with 0.01% poly-L-lysine (A-005-M Sigma-Aldrich) (10 min), followed by thorough washing with water and drying. The fixed bacterial cells were then pelleted by centrifugation (21,500 × g, 30 s), resuspended in PBS adjusted to the same pH as the growth medium and mounted onto poly-L-lysine-coated slides. After drying, the unbound bacteria were removed by rinsing with Milli-Q water. The bacterial cells were immediately subjected to microscopic observation using a Leica DM600B epifluorescence microscope (100 × oil objective lens). Images were captured with a Leica DFC350 FX digital camera interfaced with a computer running LAS AF software (version 2.0.0, Leica) for image analysis.

### Microscopy – cytoplasmic condensation

MG1655 was grown (37 °C, 200 rpm) in media (LBK, MES-KOH pH 5, 3 ml in a 30 ml tube) to mid exponential phase OD_600_ = 0.3 (NanoDrop, ND-1000). The first sample was taken before the addition of nalidixic acid and designated as the zero minutes time point. Before treatment and after 60 min incubation with antibiotic, cells were spotted onto a 1.5% agarose pad made with growth media containing nalidixic acid (150 µg/ml). Cytoplasmic condensation (CC) was monitored over 4 h starting 1 h post treatment**.** Imaging of samples were carried out on a Nikon ECLIPSE Ti2-E microscope equipped with a X-Light V3 spinning disc confocal module (CrestOptics) with a Nikon 60 × 1.42 NA Plan Apochromat λD oil objective, an objective heater, and a CO_2_ stage-top incubation chamber, maintaining samples at 37 °C. Images were taken at indicated time points using NIS-element software (Nikon) with DIC channel.

### Image analysis

Images were acquired and processed using the open-source ImageJ/Fiji software (https://fiji.sc/) 2.14.0 Java 1.8.0_322 software^32^. The objectJ plugin integrated with Fiji software was employed for manual quantification of the cell axis length with the cell counter plugin. The data were visualized with GraphPad Prism (Web site: http://www.graphpad.com/quickcalcs/ConfInterval1.cfm).

### Forward UV-induced rifampicin resistance assay

pH-adjusted PBS and LBK media were used throughout. Wild-type *E. coli* (MG1655) was grown overnight (37 °C, 1.5% agar) before being diluted and resuspended (< 1000 bacteria / culture) in liquid media for overnight growth (37 °C, 200 rpm). The following day, the bacteria were diluted (× 50) and grown to an OD_600_ of 0.25 before being centrifuged, resuspended in PBS and exposed to UV (20 J/m^2^) or not. All cultures were subsequently kept in the dark. Samples of the cultures were then plated on rifampicin (100 µg/ml) plates and on LBK to determine the CFU. The cultures were then centrifuged and resuspended in LBK before incubation (37 °C, 200 rpm, ON). The following day the cultures were plated on rifampicin (100 µg/ml) and LBK to determine the CFU. The means and standard deviations were calculated from three independent experiments.

### Numerical data

All the numerical data underlying the figures can be found in the supplementary data file.

### Statistical analysis

**Fig. 1:**
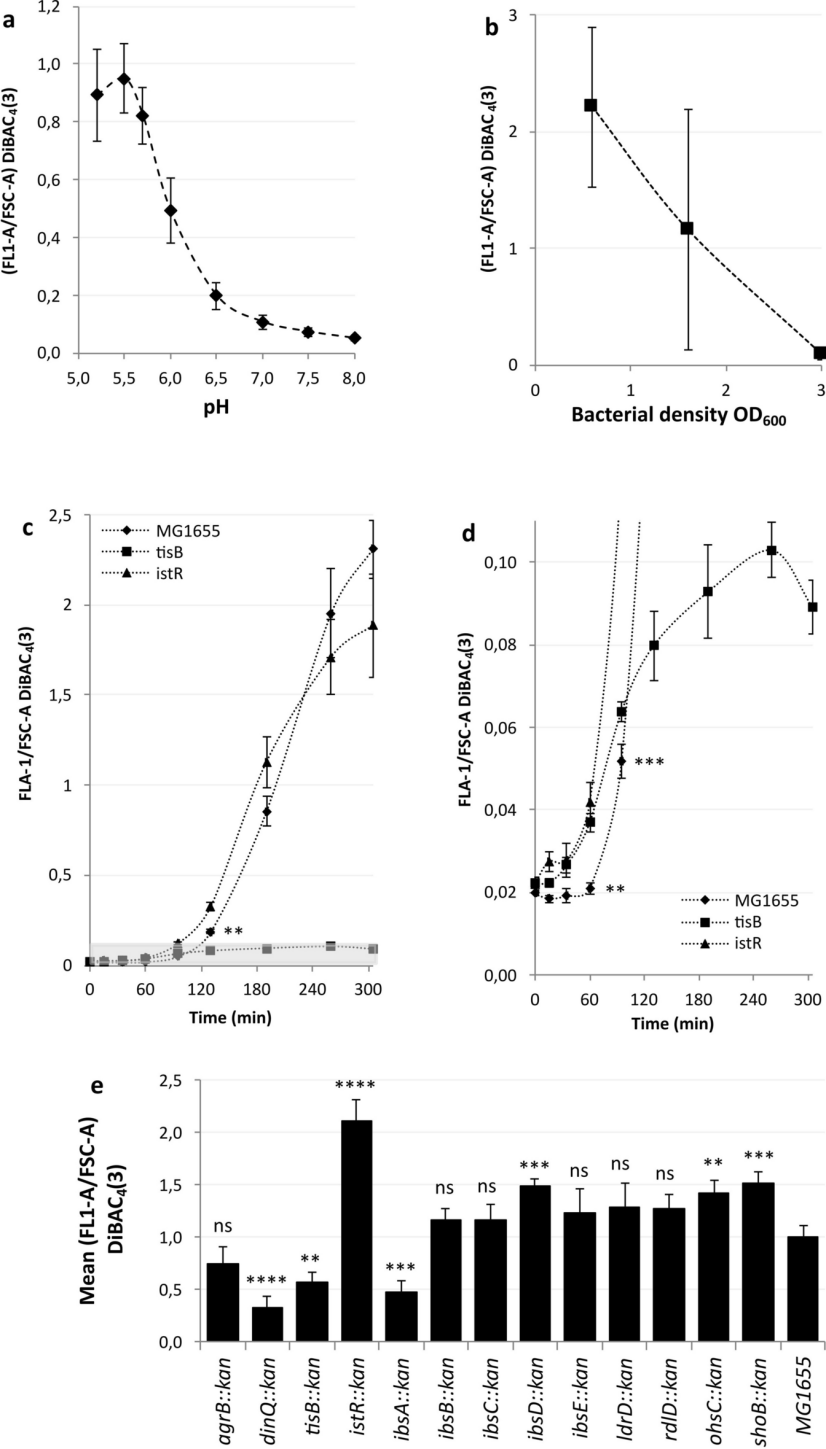
Multiple type I TA systems can result in loss of electrical potential following antibiotic-induced DNA damage. **a** DiBAC_4_(3) is most sensitive to the electrical potential at pH values of 5.2–5.7. Bacteria were grown to the exponential phase in LBK at the pH shown before being treated with 100 µg/ml nalidixic acid. The samples were further incubated for five hours before being stained with DiBAC_4_(3) in PBS buffered to the same pH as that of the media. **b** DiBAC_4_(3) is most sensitive at low bacterial densities. Bacteria were grown to the exponential phase in LBK at pH 5.7 before being treated with 100 µg/ml nalidixic acid. The samples were further incubated for five hours before staining with DiBAC_4_(3) in PBS, pH 5.7. **c-d** *tisB* and *istR* begin electrical depolarization before the wild type but achieve lower total signals after five hours, with *tisB* achieving little total depolarization. Bacteria were grown to OD_600_ = 0.6 in LBK at pH 5.7 before being treated with 100 µg/ml nalidixic acid. The samples were taken at the time points indicated and stained with DiBAC_4_(3) in PBS, pH 5.7. The gray shaded area in **c** illustrates the region of the diagram examined in **d**. The statistical significance refers to MG1655 vs *istR*. Significance for *tisB* vs *istR* was reached after 95 min and was at least p = 0.008 (Supplementary data file). Significance was calculated using a two tailed unpaired T-test. **e** Multiple type I TA mutants can result in reduced electrical depolarization. Bacteria were grown to OD_600_ = 0.6 in LBK at pH 5.7 before being treated with 100 µg/ml nalidixic acid. The samples were removed after five hours and stained with DiBAC_4_(3) in PBS, pH 5.7. All data points shown are means ± standard deviations, *n* = 3 or 4 (independent biological replicates). Statistical significance was assessed using a one-way ANOVA followed by Dunnett’s post hoc test for multiple comparisons (Supplementary data file).

All data were assumed to be normally distributed with approximately equal variances. Due to the limited number of replicates two tailed unpaired T-tests were carried out in most cases to determine statistical significance. In the case of the data underlying Fig. 1e a one-way ANOVA followed by Dunnett’s post hoc test for multiple comparisons was determined as all samples were processed under the same conditions. The numerical data can be found in the supplementary data file.

## Results

### Type I TA system mutants show altered electrical depolarization.

One of the generic phenotypes observed in many TA systems upon the overexpression of the toxin moiety is increased staining with the pH-insensitive anionic dye (bis-(1,3-dibutylbarbituric Acid)Trimethine Oxonol) DiBAC_4_(3)^4,7,33,34^. It has been demonstrated that DiBAC_4_(3) does not enter bacteria in *tisB* mutants following DNA damage^15,33^, and as many previous phenotypes have been observed across several TA systems we wanted to test whether this was the case with DiBAC_4_(3) staining following antibiotic induced DNA damage. We also investigated type I TA systems not specifically regulated by the SOS response via LexA, as little is known about their regulation. During the SOS response, genes not regulated by a LexA box continue to be expressed and function. Additionally, thousands of genes other than those regulated by a LexA box are affected by DNA damage^17,35,36^. First, we generated mutants of both the toxin and the antitoxin of systems *tisB-istR*, *ldrD-rdlD* and *shoB-ohsC*. The genetic organization of the *ibs-sib* genes, where the ORF for the *ibs* peptide is within the transcribed *sib* RNA, precluded the generation of individual mutants, so only mutants of the entire *ibs-sib* systems were created. Second, the DiBAC_4_(3) assay following nalidixic acid-induced DNA damage was optimized for maximum fluorescent signal. Preliminary studies demonstrated that pH equilibration was dependent on the bactericidal activity of nalidixic acid and that this equilibration required a minimum of 20 µg/ml nalidixic acid. A maximum effect was observed at 100 µg/ml and this concentration was subsequently used for this work. We suggest that this concentration is medically relevant as nalidixic acid reaches a peak concentration of 200 µg/ml in urine during treatment for urinary tract infections^37^. Nalidixic acid efficacy is pH dependent^16,38^, so at 100 µg/ml we used at least 5 × the minimum inhibitory concentration. Initially, we monitored the effects of pH, as we previously noted the importance of pH following antibiotic-induced DNA stress^16^ (Fig. 1a). Here, the bacteria were incubated for five hours following treatment with nalidixic acid, as we previously noted, which resulted in a strong DiBAC_4_(3) depolarization signal^16^. To balance the inhibitory effects on growth at low pH, a maximum signal pH of 5.7 was chosen. When the potentiometric cationic probe DiSC_3_(5) was used in the gram-positive species *B. subtilis* and *S. aureus,* the bacterial density was found to be critical in determining the fluorescence intensity^28^. As such, we observed that the early growth phase of *E. coli* was optimal for signal strength following antibiotic-induced DNA damage (Fig. 1b). Using these optimized parameters, we aimed to observe how the polarity of *tisB* and *istR* mutants reacted to antibiotic-induced DNA damage compared with previous results with ciprofloxacin^33^. We found that our results are broadly similar, with earlier electrical depolarization in the *istR* mutants and much weaker depolarization in the *tisB* mutant (Fig. 1c). However, we noted that the electrical depolarization observed in the *tisB* mutant was still significant and increased in strength faster than that of the background strain and as fast as that observed for the *istR* mutant within the first hour (Fig. 1d). Even after 90 min, the depolarization observed in the *tisB* mutant was equivalent to that of the wild-type background strain (Fig. 1d). We subsequently, wished to determine whether there were any type I TA systems that completely prevented depolarization. We examined all the TA mutants we created using the optimised DiBAC_4_(3) staining protocol but used a pH of 5.2 on this occasion and measured depolarization only after a five-hour incubation (Fig. 1e). Compared with the background, the *dinQ, ibsA* and *tisB* mutants all presented significantly reduced DiBAC_4_(3) staining, whereas the *istR* and *shoB* mutants presented particularly strong signals. The signals of the *ibsD*, *rdlD* and *ohsC* mutants were significantly elevated even if the signals were relatively low. Surprisingly, on this occasion, the *tisB* mutant was significantly more depolarized than at pH 5.7 (Fig. 1c & 1d), and the *istR* mutant was more depolarized than the wild type, demonstrating the pH sensitivity of depolarization in type I TA system mutants. These results confirm previous findings on the *tisB-istR* system^33^ but also demonstrate that several TA systems can affect DiBAC_4_(3) staining following antibiotic-induced DNA damage and that the level of depolarization is pH dependent. The optimization process also revealed how both the growth phase of the bacteria and the environmental pH strongly influence DiBAC_4_(3) staining.

### The tisB mutant maintains pH homeostasis, whereas the istR mutant shows accelerated loss of pH homeostasis following DNA damage

**Fig. 2:**
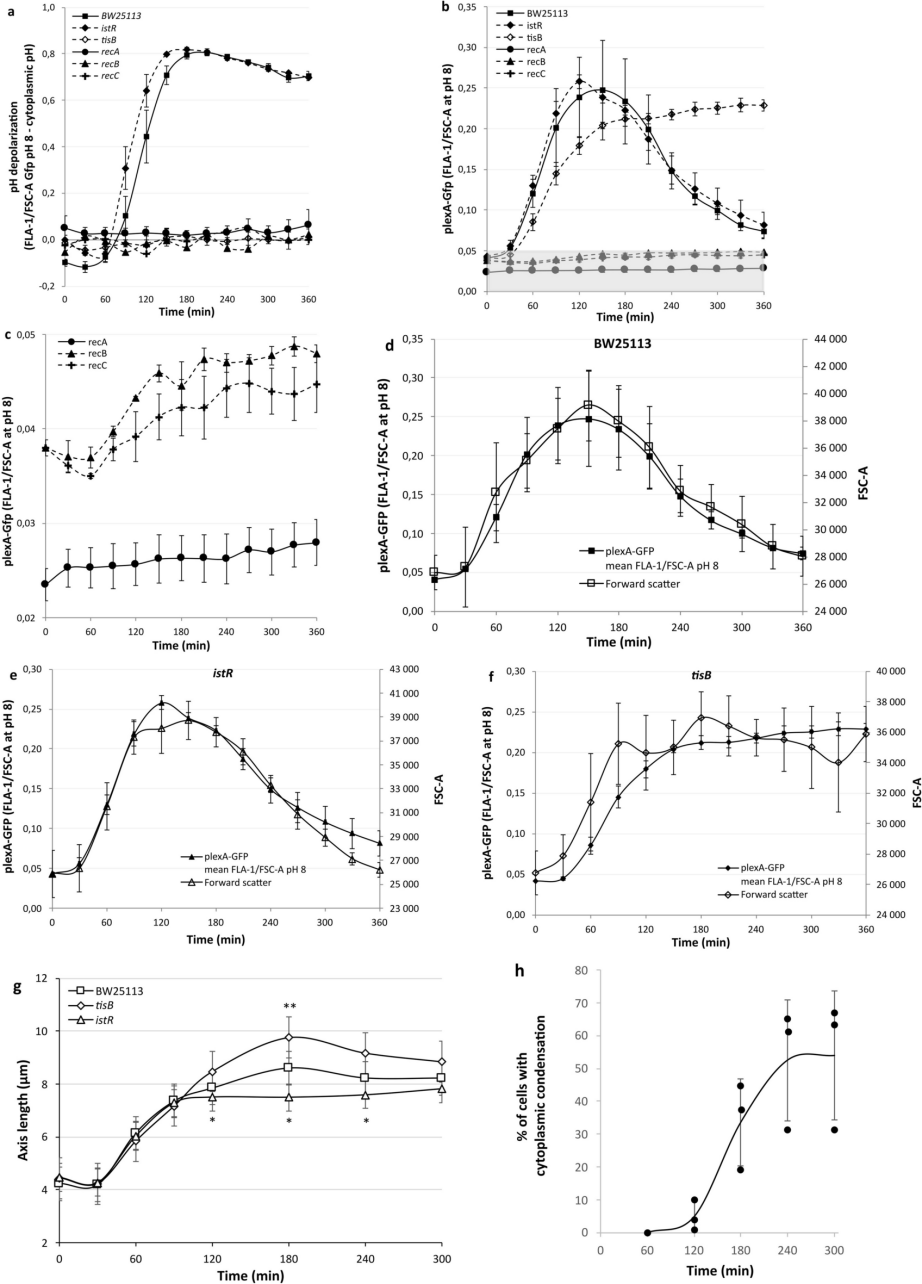
Induction of *tisB* expression affects the extent of pH depolarization and flow cytometry scattering. All strains (**a**-**f**) contained plexA-gfp and were incubated at pH 5.7 in LBK. Nalidixic acid (100 µg/ml) was added at zero minutes, resulting in immediate translation of pH-sensitive Gfp. **a** At the indicated time points, the fluorescence density of the bacteria was recorded at cytoplasmic pH and at pH 8 by depolarising the bacteria with sodium benzoate. The difference in fluorescence intensity was corrected for the amount of Gfp present as measured by fluorescence density at pH 8. No statistical significance was seen between BW25113 and *istR* using a two tailed, unpaired T-test. BW25113 was statistically significant from *tisB* at 120 min. (Supplementary data file). **b** At the indicated time points, the fluorescence density of the bacteria was recorded at pH 8 with the use of sodium benzoate. This corresponds to the activity of the *lexA* promoter. The gray shaded area in **b** illustrates the region of the diagram examined in **c**. **c** As with **b** but focusing on the reduced signals from *recA-C*. The difference between *recA* and *recB* or *recC* was statistically significant at all time points. No difference was seen between *recB* and recC using a two tailed, unpaired T-test (Supplementary data file). Increases in signal intensity were significant between 60 min, the low, and 360 min for the *recB* and *recC* mutants, p = 0.0016 and 0.0319 respectively. **d-f** Comparison of *lexA* promoter activity and the extent of forward scattering (FSC-A). **g** Comparison of the extent of filamentation as determined by microscopy. Statistical significance between *istR* and *tisB* was achieved from 120–240 min and significance between BW25113 and *tisB* at 180 min using a two tailed, unpaired T-test (Supplementary data file). **h** Kinetics of cytoplasmic condensation in wild type bacteria at pH 5, individual data points and the mean are shown. Time is the time since the addition of nalidixic acid. All data points shown are means ± standard deviations, *n* = 3 (n =  > 53 in **g**, n = 100–300 in **h**) (independent biological replicates were obtained).

Previously, we described how bacteria respond to bactericidal antibiotic-induced DNA damage by dissipating the proton gradient across their plasma membrane^16^. This process begins one hour after antibiotic treatment and full dissipation occurs after 2–3 h. This contrasts with the other component of the PMF, the electrical potential difference measured by DiBAC_4_(3) staining, which shows that depolarization still occurs even after five hours (Fig. 1c). *tisB* mutants have been implicated in DNA damage induced persister cell formation and pH depolarization^9,11,15^. We, therefore, wished to test this in our system using a *lexA* promoter Gfp protein fusion plasmid and flow cytometry^16,29^. This system allows for the simultaneous monitoring of bacterial volume, extent of DNA damage induction and durability, and proton gradient dissipation. As *tisB* is induced by the SOS response and nalidixic acid induces DNA double-strand breaks, mutants of the *recA, recB* and *recC* genes from the keio collection^39^ were included in the analysis. A low environmental pH of 5.7 was used to create a significant proton gradient across the plasma membrane for comparison with the electrical depolarization observations. As previously observed, the *tisB* mutant completely lacks the proton equilibration observed in the background strain (Fig. 2a). The SOS response mutants *recA*, *recB* and *recC,* which prevent the transcription of *tisB,* also lacked equilibration. The *istR* mutant, on the other hand, showed an accelerated induction of equilibration; however, the extent and durability of equilibration were indistinguishable from those of the background strain (Fig. 2a). The transcription of *lexA* is relatively quickly and strongly initiated in the SOS response^16^ and, as such, provides an expedient measure of how fast, intense and durable the SOS response is. We have previously shown that when the transcription of SOS genes is reduced under pH 6, the extent of this effect is determined by the temporal balance between the timing of transcription initiation and proton gradient dissipation^16^. Our hypothesis was, therefore, that a *tisB* mutant would not show any reduction in SOS as the homeostatic cytoplasmic pH is maintained. As observed previously, the background strain showed that *lexA* transcription was reduced almost to the background level after six hours of exposure to the antibiotic (Fig. 2b). The presumed origin of the loss of the Gfp signal is the decrease in the intracellular pH, which in turn reduces the activity of the *lexA* promoter, leading to a decrease in the Gfp concentration and, therefore, a decrease in the fluorescent signal. Confirming our hypothesis, a *tisB* mutant shows continuously increasing concentrations of Gfp with a reduced initial rate of induction, possibly as the initial pH reduction in the background strain results in accelerated induction^16^. The *istR* mutant presented slightly accelerated initiation and initial deactivation of SOS, with a maximum signal 30 min before that of the wild-type. Broadly, however, the *istR* mutant follows the same path as the background strain with respect to initiation, maximum expression, and deactivation. As expected, the *recA* mutant presented no increase in Gfp; however, both the *recB* and *recC* mutants presented a significant increase in signal intensity (Fig. 2c). More surprisingly, the lack of signal reduction at later time points was a pattern that was also observed in the *tisB* mutant. In addition to fluorescence measurements, flow cytometry was used to quantify the degree of scattering produced by the particles. Bacterial scattering is dependent on size, shape and their refractive index^40,41^. The refractive index of bacteria, in turn, can be dependent on the cell wall composition, internal structure and environmental factors^42^. During our experiments, we observed a strong association between the transcriptional activity of the *lexA* promoter and forward scatter for the wild type and all single deletion mutants we observed (Fig. 2d-f and supplementary Fig. S2a-c). These findings suggest a link between bacterial physical properties and antibiotic-induced DNA damage.

Bacterial scattering in flow cytometry has been associated with cell volume^40^. A surprising decrease in the scattering of the wild type and *istR* mutant led us to examine the bacteria using microscopy following antibiotic-induced DNA damage to determine if filamentation was decreasing (Fig. 2g and supplementary Fig. S3). We found that filamentation increased for up to 180 min for the wild type and *tisB* mutant before decreasing slightly, whereas filamentation in the *istR* mutant stopped increasing at 120 min and then maintained this degree of filamentation. The decrease in the scattering readings, therefore, appears to be the result of a physical characteristic other than bacterial length. Interestingly, filamentation after 90 min was *tisB* specific, with an excess of *tisB* resulting in a rapid stop in filamentation, whereas the wild-type levels slowly increased, and the absence of *tisB* led to the highest levels of filamentation.

Prior studies have demonstrated that fluoroquinolone treatment induces cytoplasmic condensation^15,43^.Given this established relationship, we investigated the impact of an acidic environment on both the frequency and temporal dynamics of cytoplasmic condensation in exponentially growing wild-type bacteria exposed to nalidixic acid. Our results reveal that while cytoplasmic condensation still occurs under low pH conditions, the phenomenon affects approximately a third fewer cells and exhibits a 60-min delay in onset compared to previous observations under standard conditions (Fig. 2h and Supplementary Fig. S4).

### *tisB* is primarily responsible for proton gradient dissipation

**Fig. 3:**
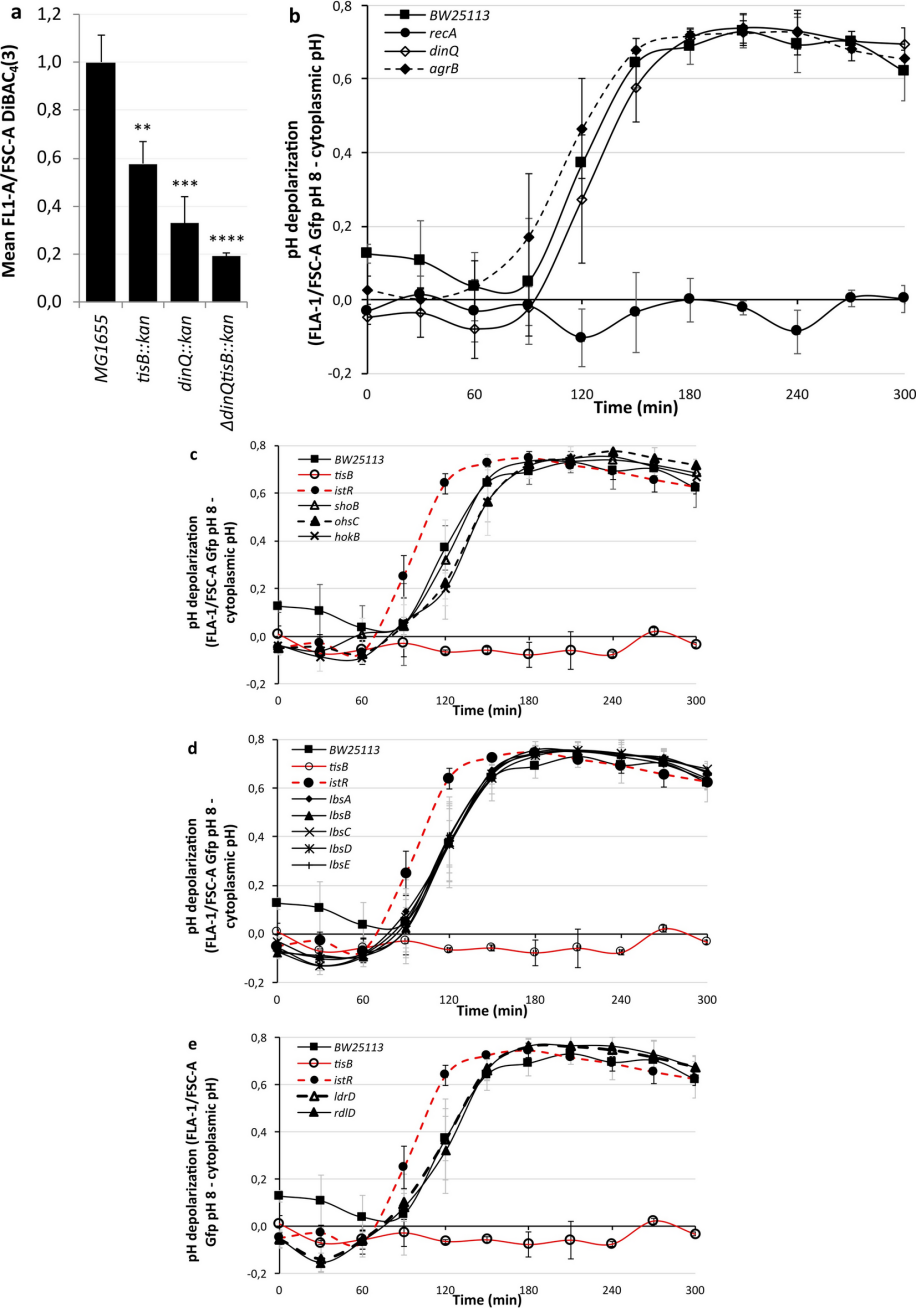
*tisB* is responsible for pH depolarization. In all cases, the strains were grown to the exponential phase in LBK before being exposed to 100 µg/ml nalidixic acid. **a** A double *dinQ tisB* mutant results in significantly less DiBAC_4_(3) staining than either *dinQ* or *tisB* alone following antibiotic-induced DNA damage. The fluorescence density measurements of bacteria stained with DiBAC_4_(3) was measured on a flow cytometer after five hours of incubation with nalidixic acid. A two tailed, unpaired T-test was used to assess significance (Supplementary data file). **b-e** Differences in the fluorescence density measurements of bacteria containing Gfp at the intracellular pH and at pH 8, through the use of the weak acid sodium benzoate, were calculated at the time points indicated. **b** *dinQ* and *agrB* mutants varied only slightly from the wild type rate of proton gradient dissipation whilst *recA* mutants did not equilibrate. **c** *tisB* mutants did not dissipate the proton gradient, whereas *istR* mutants presented an accelerated rate of dissipation compared with the wild-type. Compared with those of the wild type, the proton gradient dissipation rates of the *shoB*, *ohsC* and *hokB* mutants did not differ. **d** None of the ibsA-E mutants deviated from the wild-type rate of proton gradient dissipation. **e** Neither *ldrD* nor the *rdlD* mutants showed any difference in rate from the wild-type pH equilibration rate. All data points shown are means ± standard deviations, *n* = 4 **a,** 5 **b-e** (independent biological replicates).

*E. coli* has three LexA-sensitive type I TA systems, *dinQ-agrB*, *tisB-istR* and *symE-symR*. SymE, previously thought to be an intracellular RNase^44^ and now shown to be a DNA-binding protein^45^ is found intracellularly. *dinQ* and *tisB* produce small hydrophobic peptides that localize to the inner membrane and mutants of *tisB* reduce the extent to which the bacterium takes up DiBAC_4_(3) staining following DNA damage^15,33^. As such, we asked the following question: are *tisB* and *dinQ* in the same pathway responsible for DiBAC_4_(3) staining, or do they have additive effects, as they play different roles in ion transport? We constructed a double mutant lacking *dinQ* and *tisB* and demonstrated a significant additive effect on staining at pH 5.2, suggesting differing roles in the depolarization of the electrical gradient (Fig. 3a). As both the *dinQ* and *tisB* mutants result in a significant reduction in DiBAC_4_(3) staining following DNA damage we hypothesized that both genes also affect proton depolarization. To confirm this, we carried out a proton dissipation experiment with the *recA*, *dinQ* and *agrB* mutants with the plexA-gfp system. Surprisingly, the results demonstrated that deletion of neither *dinQ* nor *agrB* resulted in a significant reduction in proton gradient dissipation. *dinQ* was almost indistinguishable from the background strain, whereas *agrB* dissipated the proton gradient marginally earlier than the wild type did (Fig. 3b). Earlier experiments (Fig. 1c) demonstrated that several other mutants from TA systems affect the extent of DiBAC_4_(3) staining following DNA damage. To investigate whether any of these systems affect proton gradient dissipation, we monitored the response of these mutants following antibiotic-induced DNA damage. On this occasion, we included *hokB* from the Keio collection, a type I TA system known to form persister state bacteria^46^. None of the tested type I TA system mutants showed any effect on proton gradient dissipation (Fig. 3c-e), suggesting that *tisB* is solely responsible for this physiological response to DNA damage in almost all circumstances.

### The proton and electrical gradients are differentially dissipated over time

**Fig. 4:**
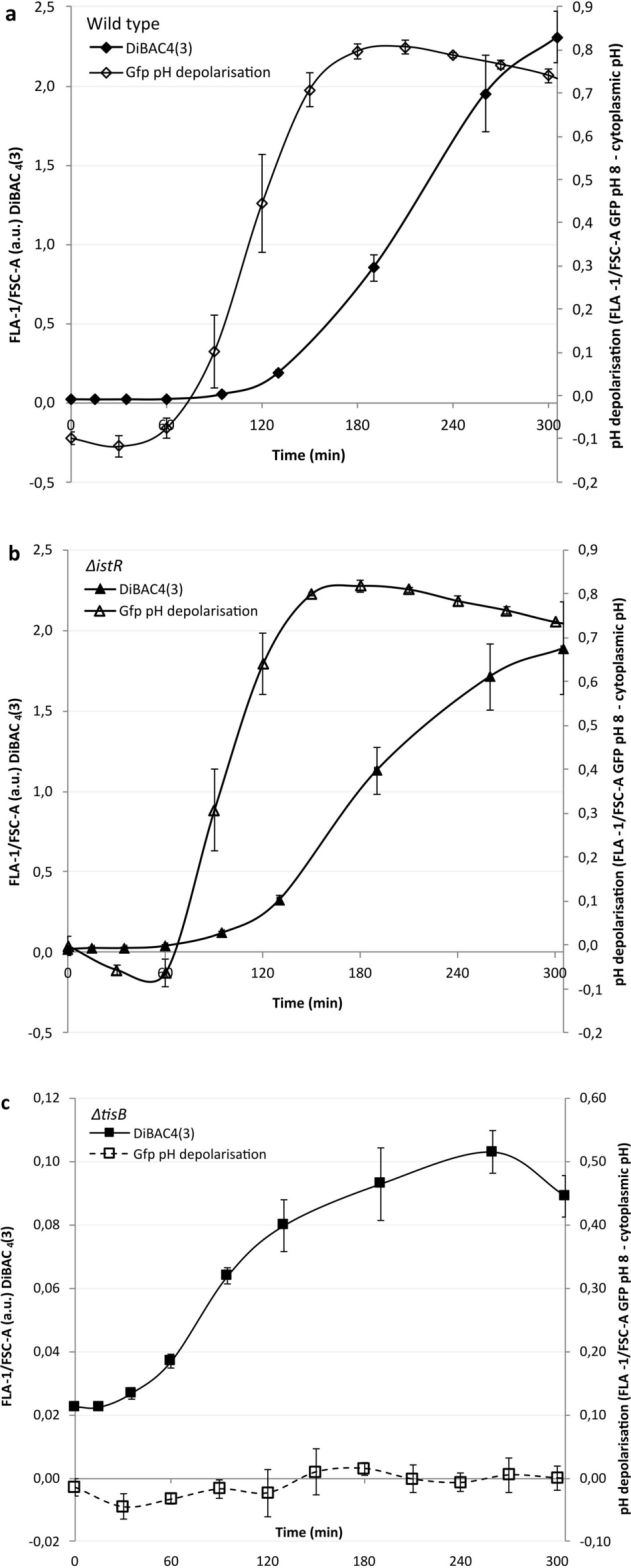
The pH gradient dissipates faster than the electrical potential. In all cases, exponentially growing strains in LBK buffered to pH 5.7 were exposed to 100 µg/ml nalidixic acid at time = zero minutes. Fluorescence density measurements of either DiBAC_4_(3) to measure the electrical potential (closed symbols) or Gfp to measure the pH gradient (open symbols) were monitored using flow cytometry at the time points indicated. The pH gradient is the difference in fluorescence intensity at the intracellular pH versus that at pH 8 divided by the amount of Gfp present. **a** Wild-type *E. coli*. **b** *istR* deletion mutants show faster depolarization of both potentials as compared to the wild type and **c** *tisB* deletion mutants show no proton depolarization but fast initial electrical depolarization compared with the wild type, which fails to accelerate after 60 min. All data points shown are the means ± standard deviations, *n* = 4 or 5 (independent biological replicates).

The PMF is composed of a chemical or proton gradient and an electrical gradient. Owing to identical experimental setups, the kinetics of electrical depolarization and proton gradient dissipation can be compared. The differing kinetics indicate that the dissipation events are mechanistically distinct (Fig. 4a). The proton gradient in the wild type remains approximately constant for the first 90 min before abruptly dissipating over 60 min on average and is then approximately stable for the remainder of the experiment. In contrast, the electrical potential remains constant for the first 60 min before gradually increasing, and the majority of the change then occurs over two hours; however, even after five hours, the gradient still appears to be dissipating. The *istR* mutant shows earlier depolarization of both gradients (Fig. 4b). Additionally, electrical depolarization occurs after only 15 min and gradually accelerates thereafter, but as observed with the wild type, dissipation occurs over the five hours in which the observations were carried out. The *tisB* mutant shows no proton gradient dissipation and a small electrical depolarization, which similar to the *istR* mutant, begins early but reaches a maximum after approximately four hours of incubation (Fig. 4c). Taken together, these comparisons show that all strains will have access to differing amounts of PMF, and even in the case of the *istR* mutant with accelerated depolarization rates, some residual PMF may still exist even after five hours.

### Loss of *tisB* results in increased expression of *umuDC* and increased rates of mutagenesis

**Fig. 5:**
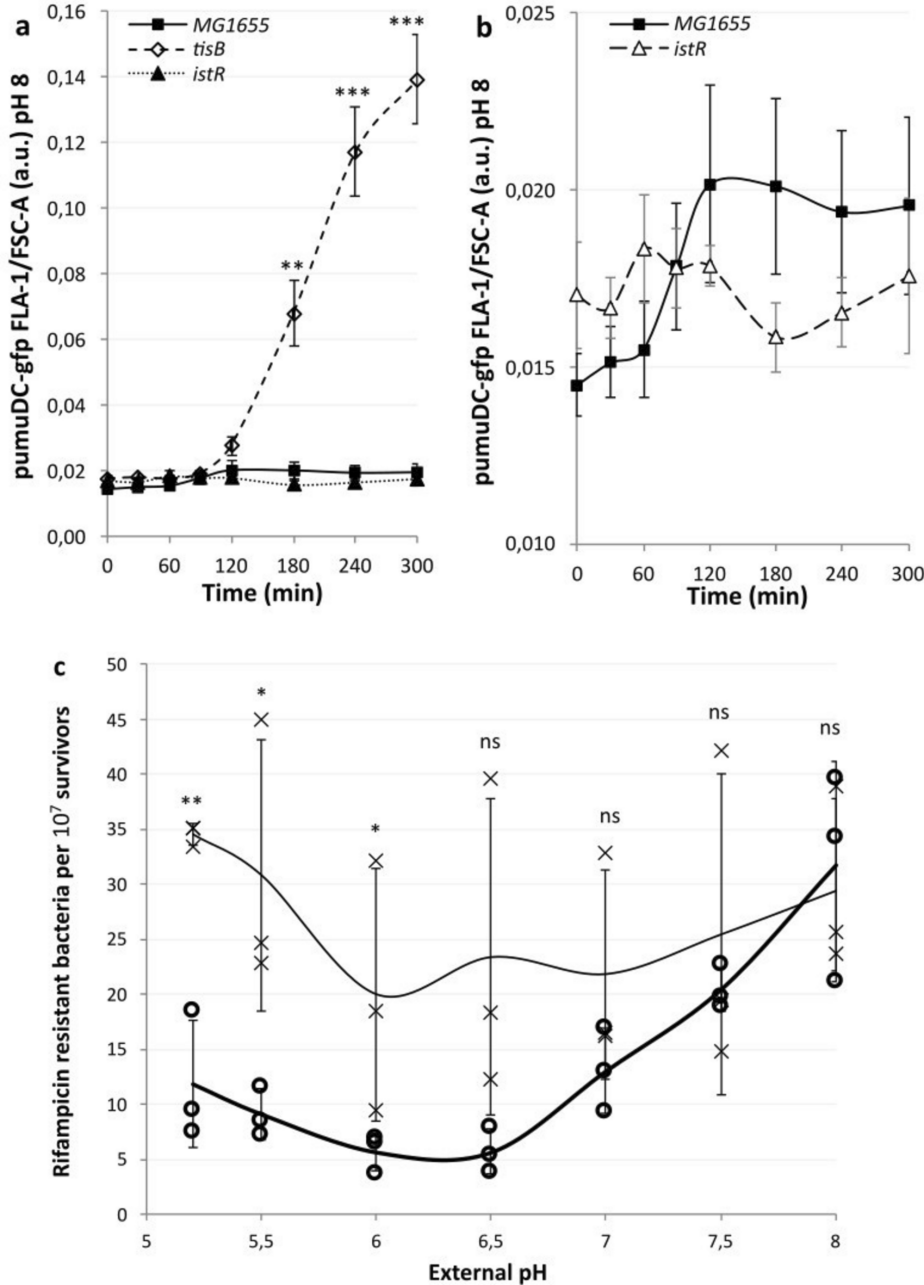
Failure of *tisB* mutants to depolarize results in elevated *umuDC* promoter activity and mutagenesis in low pH environments. The rate of proton depolarization in low-pH environments determines the extent to which *umuDC* transcription is inhibited. **a** The *tisB* mutant, which cannot depolarise the proton gradient, results in large increases in *umuDC* compared with the background strains. Flow cytometry measurements are of the GFP fluorescence density of a umuDC promoter GFP construct, over time. Exponentially growing cultures at pH 5.7 were treated at 0 min with 100 ug/ml nalidixic acid to induce the SOS response. All fluorescence readings were quantified at pH 8. A two tailed, unpaired T-test was used to assess significance (Supplementary data file). **b** The *istR* mutant, with an accelerated rate of depolarization, led to no increase in fluorescence density, whereas the wild type presented a small but not significant increase (Supplementary data file). **c** Increased mutagenesis is observed in the *tisB* mutant, a cross marker, at low external pH compared with the wild type, open circle marker. Exponentially growing cultures were exposed to UV light (20 J) before further growth and subsequent plating on rifampicin plates (100 µg/ml). A two tailed, unpaired T-test was used to assess significance (Supplementary data file). All data points shown are means ± standard deviations, *n* = 3 or 4 (independent biological replicates).

Previously, we reported that the *umuDC* operon is induced following the dissipation of the pH gradient and that the promoter activity of *umuDC* is pH dependent, with startlingly little activity at pH 5.2^16^. As such, we expected the promoter activity of *umuDC* in a *tisB* mutant, where we expect the intracellular pH to be maintained at homeostatic levels, to be considerably stronger than that of the wild type in acidic environments. To explore this hypothesis, we produced strains that contained the pumuDC-gfp plasmid (Supplementary Table S1) and followed Gfp expression following antibiotic-induced DNA damage by flow cytometry (Fig. 5a). The maintenance of homeostatic pH in the *tisB* mutant ultimately led to a sevenfold increase in Gfp expression compared with that in both the background strain MG1655 and the *istR* mutant. However, the MG1655 background strain presented a small but significant increase in Gfp, yet the *istR* mutant presented in change (Fig. 5b). Previous findings have shown that the overexpression of *tisB* results in decreased mutagenesis^10^. UmuC and UmuD are essential components of the mutagenic translesion polymerase PolV. Using a UV-induced rifampicin resistance assay, we examined whether the increased activity of the *umuDC* promoter ultimately led to increased mutagenesis. As previously reported, the extent of mutagenesis is dependent on the pH of the external media, so mutagenesis was examined over the viable pH range for *E. coli*^16^. As anticipated, the *tisB* mutant led to increased levels of mutagenesis at low pH (Fig. 5c). The difference between *tisB* and the background decreased as the pH increased, with no differences observed at or around homeostatic pH.

Taken together, these results suggest that *tisB* is specifically responsible for the dissipation of the proton gradient following the induction of the SOS response. Increased concentrations of TisB produced by the dysregulation of translation in an *istR* antitoxin deletion mutant lead to accelerated proton gradient dissipation. The results from the other type I TA systems investigated here show that they can affect the electrostatic potential but do not contribute to the dissipation of the proton gradient.

## Discussion

Following a lethal antibiotic shock *E. coli* depolarize not only their electrical gradient but also the proton gradient, the two components of the PMF^16^. *E. coli* maintains a homeostatic pH of approximately 7.6 and is viable over an external pH range of 5–9^47^. Owing to the constraint of homeostatic pH, the proton and electrical gradients vary over the viable environmental pH range to maintain a PMF of approximately -170 mV^48^. The generation of persister state bacteria in which the PMF and ATP concentrations are reduced or eliminated^11,49^ over the viable environmental pH range requires that both electrical and proton gradients be dissipated. Elimination of one gradient can lead to compensatory increases in the other PMF component^50^, so it might be necessary for both gradients to be equilibrated simultaneously.

Here, we show that several type I TA systems can be involved in the dissipation of the electrical gradient following nalidixic acid-induced DNA damage. The electrical gradient is the sum of many complex charge gradients^51^, and as such, we hypothesize that each TA system may be responsible for the depolarization of specific components of the electrical gradient. Our results suggest that the depolarization seen with the double *dinQ* and *tisB* mutant strains compared with either single mutant strain shows that these genes have synergistic effects with respect to the total electrical transmembrane potential. A synergistic role in persister cell formation between *tisB* and another component of the RecA regulon has previously been demonstrated^11^. In contrast to the electrical gradient, we confirm that among all the type I TA systems we examined, only *tisB* appears to facilitate the proton equilibration. The chemical gradient component of the PMF also contrasts with the electrical gradient in that it is simply defined as the gradient of protons across the plasma membrane in bacteria, possibly allowing for this gradient to be controlled by a single gene product.

Recent work illuminates the complex relationship between TisB and persister formation^15^. Previously TisB’s contribution to fluoroquinolone persistence was shown to be highly condition-dependent^52^. While *∆tisAB* mutants showed wild-type survival levels under their standard conditions, they exhibited a 25-fold reduction in survival after 5 h of ciprofloxacin treatment when tested under conditions matching previous studies^11^. These findings suggest that environmental factors may substantially modulate the SOS response’s role in persistence, potentially explaining disparate results across different experimental settings.

**Fig. 6:**
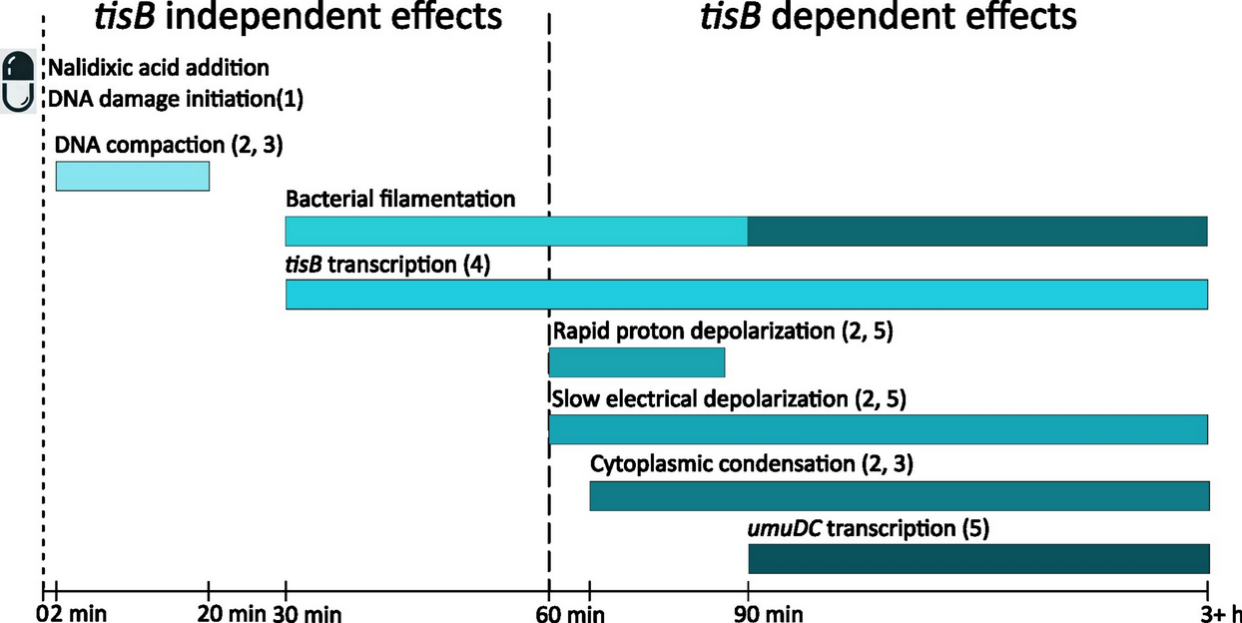
TisB dependent and Associated Cellular Events Following DNA Damage in *E. coli.* The addition of nalidixic acid, a DNA-damaging antibiotic, triggers a cascade of cellular events through inhibition of DNA gyrase and topoisomerase IV. This inhibition leads to blocked replication and transcription which ultimately results in DNA double-strand breaks^62^. These breaks are processed by RecBCD, leading to RecA loading onto ssDNA. The resulting nucleoprotein filament catalyzes LexA autodegradation, activating the SOS response. Cellular responses occur in a defined temporal sequence: DNA compaction initiates within minutes and peaks at 20 min post-treatment^15,63^. Cell filamentation, driven by SulA-mediated FtsZ inhibition, begins at 30 min and continues to increase for up to 3 h (Fig. 2g). *tisB* transcription begins to increase after 30 min^64^. TisB-dependent effects emerge at 60–90 min post-treatment, coinciding with membrane depolarization (disruption of electrical (Fig. 1c) and proton gradients (Fig. 2a)^15,16^) and subsequent cytoplasmic condensation (Fig. 2h). Filamentation becomes *tisB* dependent at this stage (Fig, 2g). At later stages the *umuDC* promoter transcription levels are highly *tisB* dependent (Fig. 5a). These events can lead to bacterial persistence under specific conditions^11,52^. While the presented timings are indicative and illustrative, the relative ordering of events is expected to be maintained. Observed timings will vary depending on environmental conditions and whether bulk or single-cell readings are taken.

Our observations partially align with an earlier report^15^, particularly regarding the timing of initial membrane potential changes, which begin approximately one hour after treatment in both studies. However, while they observed rapid proton depolarization completing within 15 min before cytoplasmic condensation in MOPS minimal media, our experiments in LB medium revealed a more gradual process extending over two hours. These distinct kinetics likely reflect the different growth conditions used in each study, highlighting the importance of media composition in membrane potential dynamics during the DNA damage response (Fig. 6).

Taken together, our results indicate that these two type I TA systems could work in concert to deplete the PMF by dissipating both the proton gradient and the electrical gradient. While *tisB* appears to almost completely control the dissipation of the proton gradient, *dinQ* has a greater effect on the electrical gradient. However, which components of the electrical gradient control the other type I TA systems remains to be discovered. The leading candidates include Na^+^, K^+^, Cl^-^, Ca^2+^, and Mg^2+^, which are the major contributors to intracellular ionic strength. Although the concentrations of these ions can be determined in specific media at a particular point in time^53^, concentrations vary due to osmoregulation^54^. Examples of these ions that create action potentials include potassium cations, which normally have a higher intracellular concentration than the extracellular media which can be used to hyperpolarize bacteria under specific circumstances^55,56^. Other examples include free dicationic calcium influx during voltage depolarization^57^ and chloride release to protect against acid stress^58^.

Ultimately, the PMF is a composition of many charged components. In addition, the asymmetric distribution of small permeable ionic species, large impermeable ionic molecules, and their proximity to the membrane, for example, the positioning of anionic phospholipid head groups at the membrane surface can create a high concentration of negative charges in low-cation environments. As such, polyanionic nucleic acids may play a significant role in polarity across the inner membrane^51^. Under exponential growth the genome is in a relaxed state and fills much of the space inside the bacterium. Additionally, transcription occurs at a significant rate, producing many RNAs. Following antibiotic-induced DNA damage, the genome undergoes *recN*-dependent compaction^59,60^, a process that is in part influenced by *dinQ* and *agrB*^4^. Compaction alters the distribution of DNA in contact with the membrane^59^, potentially altering the local electrical polarity; simultaneously, the number of transcripts is reduced due to RNase activity^44^, altering the distribution of charge. The redistribution of nucleic acids together with the reorganization of DNA could lead to a reduction in the plasma membrane potential locally and globally. DNA compaction occurs rapidly after the introduction of even modest DNA damage, reaching a maximum after 15 min, well before either significant chemical or electrical depolarization is detectable. As such, these rapid initial changes in the membrane potential could provide a stimulus to trigger larger global changes in polarity.

The decrease in the *lexA* promoter induced Gfp levels after 120 min of exposure to the antibiotic at low environmental pH in the BW25113 wild-type, which corresponds with previous findings^16^. The reduced intensity of the maximum signal observed here could presumably be due to the different background strain MG1655 being used previously. This reduction suggests reduced autoproteolytic degradation of LexA, which could be due to earlier findings indicating conformational changes in the RecA-ssDNA filament at pH 5^16^ or conformational changes in LexA that prevent degradation such that renewed repression of the SOS operon occurs^61^. We also found that the light scattering properties of bacteria follow those of *lexA* promoter activity. The initial component of this association is likely the filamentation of the bacteria; however, the subsequent loss of scattering in BW25113 and *istR* mutant indicates other physical changes in the bacteria or cell lysis^40^.

In conclusion, we confirm that *tisB* is exclusively responsible for the dissipation of the proton gradient following antibiotic induced DNA damage. The complexity of the electrical gradient, the other component of the PMF, is also reflected in the fact that several TA systems, including *tisB*, appear to be responsible for its regulation. Moreover, our results suggest that the LexA-sensitive type I TA toxins *dinQ* and *tisB* work cooperatively to relieve asymmetric charge distributions during antibiotic-induced DNA damage. We also found that *tisB* influences the extent of filamentation, with excess *tisB* in an *istR* mutant inhibiting filamentation, whereas the lack of *tisB* increases the extent of filamentation. Finally, we suggest that *tisB* initiates persister cell formation, under certain conditions, following the hierarchical processes initiated by DNA damage.

## Supplementary Information

Supplementary Information 1.

Supplementary Information 2.

## Data Availability

The source data underlying all the figures are included in the Supplementary Data file. Please address all inquiries about the data to J. A. Booth.
