# Supplementary material for: Escherichia coli type I toxin TisB exclusively controls proton depolarization following antibiotic induced DNA damage
Source: Sci Rep. 2025 Apr 14;15:12774. doi: 10.1038/s41598-025-96136-x (PMC11997105; doi:10.1038/s41598-025-96136-x)
Supplement: Supplementary file 1 — Supplementary Information 1. [file 41598_2025_96136_MOESM1_ESM.pdf]

Supplementary Information

Escherichia coli type I toxin TisB exclusively controls proton depolarization following antibiotic induced DNA damage.

Tekle Airgecho Lobie[1,2,3], Charlotte Solum Krog[1], Kirsten Skarstad[1], Magnar Bjørås[1,2,3,*] and James Alexander Booth,[1,2,*]

[1]Department of Microbiology, University of Oslo, and Oslo University Hospital, Rikshospitalet, Oslo, Norway

[2]Department of Clinical and Molecular Medicine, Norwegian University of Science and Technology, Trondheim, Norway.

[3]Centre for Embryology and Healthy Development, University of Oslo, Oslo 0373, Norway.

*Corresponding Authors (james.booth@ntnu.no & magnar.bjoras@ntnu.no)

Table S1. Strains and plasmids used in this study

Strain	Genotype / plasmid	Parent strain	Source
MG1655	*F-, λ-, rph-1*	-	
BW25113	*Δ(araD-araB)567, ΔlacZ4787(::rrnB-3), λ-, rph-1, Δ(rhaD-rhaB)568, hsdR514*	-	
BW25113 pSIM6	/ *pSIM6*	BW25113	Electroporation of plasmid from [27]
dinQ	*dinQ::kan*		[4]
agrB	*agrB::kan*		[4]
tisB	*tisB::kan*		pSIM6 recombineering with 18298-18299
istR	*istR::kan*		pSIM6 recombineering with 18300-18301
shoB	*shoB::kan*		pSIM6 recombineering with 18306-18307
ohsC	*ohsC::kan*		pSIM6 recombineering with 18308-18309
ldrD	*ldrD::kan*		pSIM6 recombineering with 18310-18311
rdlD	*rdlD::kan*		pSIM6 recombineering with 18312-18313
ibsA	*ibsA-sibA::kan*		pSIM6 recombineering with 19988-19989
ibsB	*ibsB-sibB::kan*		pSIM6 recombineering with 19990-19991
ibsC	*ibsC-sibC::kan*		pSIM6 recombineering with 19992-19993
ibsD	*ibsD-sibD::kan*		pSIM6 recombineering with 19994-19995
ibsE	*ibsE-sibE::kan*		pSIM6 recombineering with 19996-19997
recA	*ΔrecA*	MG1655	P1 transduction from JW2669-1 [38] and pCP20 removal of Kan^R
recA plexA-gfp	/ plexApromoter-gfpmut-2	*ΔrecA*	Electroporation of plasmid from [29]
recB	*ΔrecB*	MG1655	P1 transduction from JW2669-1 [38] and pCP20 removal of Kan^R
recB plexA-gfp	/ plexApromoter-gfpmut-2	*ΔrecB*	Electroporation of plasmid from [29]
recC	*ΔrecC*	MG1655	P1 transduction from JW2669-1 [38] and pCP20 removal of Kan^R

recC plexA-gfp	/ plexApromoter-gfpmut-2	*ΔrecC*	Electroporation of plasmid from [29]
tisB plexA-gfp	/ plexApromoter-gfpmut-2	*ΔtisB*	pCP20 removal of Kan^R and electroporation of plasmid from [29]
istR plexA-gfp	/ plexApromoter-gfpmut-2	*ΔistR*	pCP20 removal of Kan^R and electroporation of plasmid from [29]
dinQ tisB::kan	*ΔdinQ tisB::kan*	*ΔdinQ*	P1 transduction from *tisB::kan*
dinQ plexA-gfp	/ plexApromoter-gfpmut-2	*ΔdinQ*	pCP20 removal of Kan^R and electroporation of plasmid from [29]
agrB plexA-gfp	/ plexApromoter-gfpmut-2	*ΔagrB*	pCP20 removal of Kan^R and electroporation of plasmid from [29]
shoB plexA-gfp	/ plexApromoter-gfpmut-2	*ΔshoB*	pCP20 removal of Kan^R and electroporation of plasmid from [29]
hokB plexA-gfp	/ plexApromoter-gfpmut-2	*JW5225*[38]	pCP20 removal of Kan^R and electroporation of plasmid from [29]
ibsA plexA-gfp	/ plexApromoter-gfpmut-2	*ΔibsA*	pCP20 removal of Kan^R and electroporation of plasmid from [29]
ibsB plexA-gfp	/ plexApromoter-gfpmut-2	*ΔibsB*	pCP20 removal of Kan^R and electroporation of plasmid from [29]
ibsC plexA-gfp	/ plexApromoter-gfpmut-2	*ΔibsC*	pCP20 removal of Kan^R and electroporation of plasmid from [29]
ibsD plexA-gfp	/ plexApromoter-gfpmut-2	*ΔibsD*	pCP20 removal of Kan^R and electroporation of plasmid from [29]
ibsE plexA-gfp	/ plexApromoter-gfpmut-2	*ΔibsE*	pCP20 removal of Kan^R and electroporation of plasmid from [29]
ldrD plexA-gfp	/ plexApromoter-gfpmut-2	*ΔldrD*	pCP20 removal of Kan^R and electroporation of plasmid from [29]
rdlD plexA-gfp	/ plexApromoter-gfpmut-2	*ΔrdlD*	pCP20 removal of Kan^R and electroporation of plasmid from [29]
tisB pumuDC-gfp	/ pumuDCpromoter-gfpmut-2	*ΔtisB*	Electroporation of plasmid from [29]

istR pumuDC-gfp	/ pumuDCpromoter-gfpmut-2	*ΔistR*	Electroporation of plasmid from [29]

Table S2. Oligonucleotides used in recombineering. The lower-case sequences are specific to the sequence found in *E. coli* immediately adjacent to the genetic region to be removed. The sequences in upper case correspond to those found in pKD13 and are adjacent to the region containing the FRT flanking the kanamycin resistance gene.

Number	Sequence
18298	cagcggaaaggtacgtcagctggcagtgctcctgaaccaATTCCGGGGATCCGTCGACC
18299	gcggtttcccgctcccctttggtgcgacttgaatctgaaGTGTAGGCTGGAGCTGCTTC
18300	ctcaactatgcactaaatacgtcaaaattcgtgccgaaaATTCCGGGGATCCGTCGACC
18301	gagcgaggtttcgtcagtcgcctgcggctggtaaccgcaGTGTAGGCTGGAGCTGCTTC
18306	agatgtaagggctaatctgaatggctgcattccttgtttATTCCGGGGATCCGTCGACC
18307	actggttcaccggacgcaccttaagtacgtttccttgtgGTGTAGGCTGGAGCTGCTTC
18308	cgtaaaggctcgagtttttatgctaaagattgcaagttgATTCCGGGGATCCGTCGACC
18309	actggtaaggaacgggggttttactttttaactttaattGTGTAGGCTGGAGCTGCTTC
18310	ccactcaagaatagccgcgaaacgttgtcattacaacacATTCCGGGGATCCGTCGACC
18311	caaattgccattacggcagcctgacgcccgcatgacacgGTGTAGGCTGGAGCTGCTTC
18312	cctggtggcattggttgctggagagagaaaacccccgcaGTGTAGGCTGGAGCTGCTTC
18313	ggccatgcccagctctgcgaacgtcatatagccgcctgtATTCCGGGGATCCGTCGACC
19988	ggaggtaagccgatgatttcagcgggacgctgaaacgggGTGTAGGCTGGAGCTGCTTC
19989	aattttgctattcgcctcgcaagaacggagagccgtcgaATTCCGGGGATCCGTCGACC
19990	ggaggtaagccgacgatttcagcgggacgctgaaacgggGTGTAGGCTGGAGCTGCTTC
19991	atatttgctggcaggatcgcagactacaaagcctgcggaATTCCGGGGATCCGTCGACC
19992	gggatttacgatggcagggcagcatggggctgtaacggtGTGTAGGCTGGAGCTGCTTC
19993	tctgggagtggtaagggcgatacacccgcatcgccctgaATTCCGGGGATCCGTCGACC
19994	tcatcaacaatcggggagtcagcagggggctgaaacgggGTGTAGGCTGGAGCTGCTTC
19995	cggctttttgggccagggatatgtgttaatgaaaccttcATTCCGGGGATCCGTCGACC

19996	ctcatcaacaatcggggggcagcaaggggctgaaacgggGTGTAGGCTGGAGCTGCTTC
19997	aagcggttcacagatctcgcttcgtacatggcaggcctaATTCCGGGGATCCGTCGACC

Fig. S1: Gating of a typical *E. coli* population.

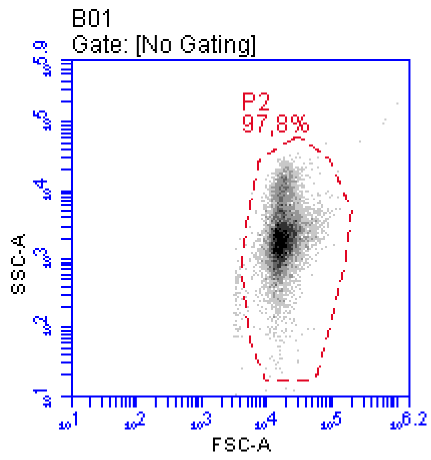

A typical gate around a typical wild-type *E. coli* population is used to remove background noise from the AccuriC6 near the threshold, and any extreme outliers liable to be debris. The threshold is set to 15000 on FSC-H.

Fig. S2: Kinetics of *lexA* induction follow those of flow cytometry scattering.

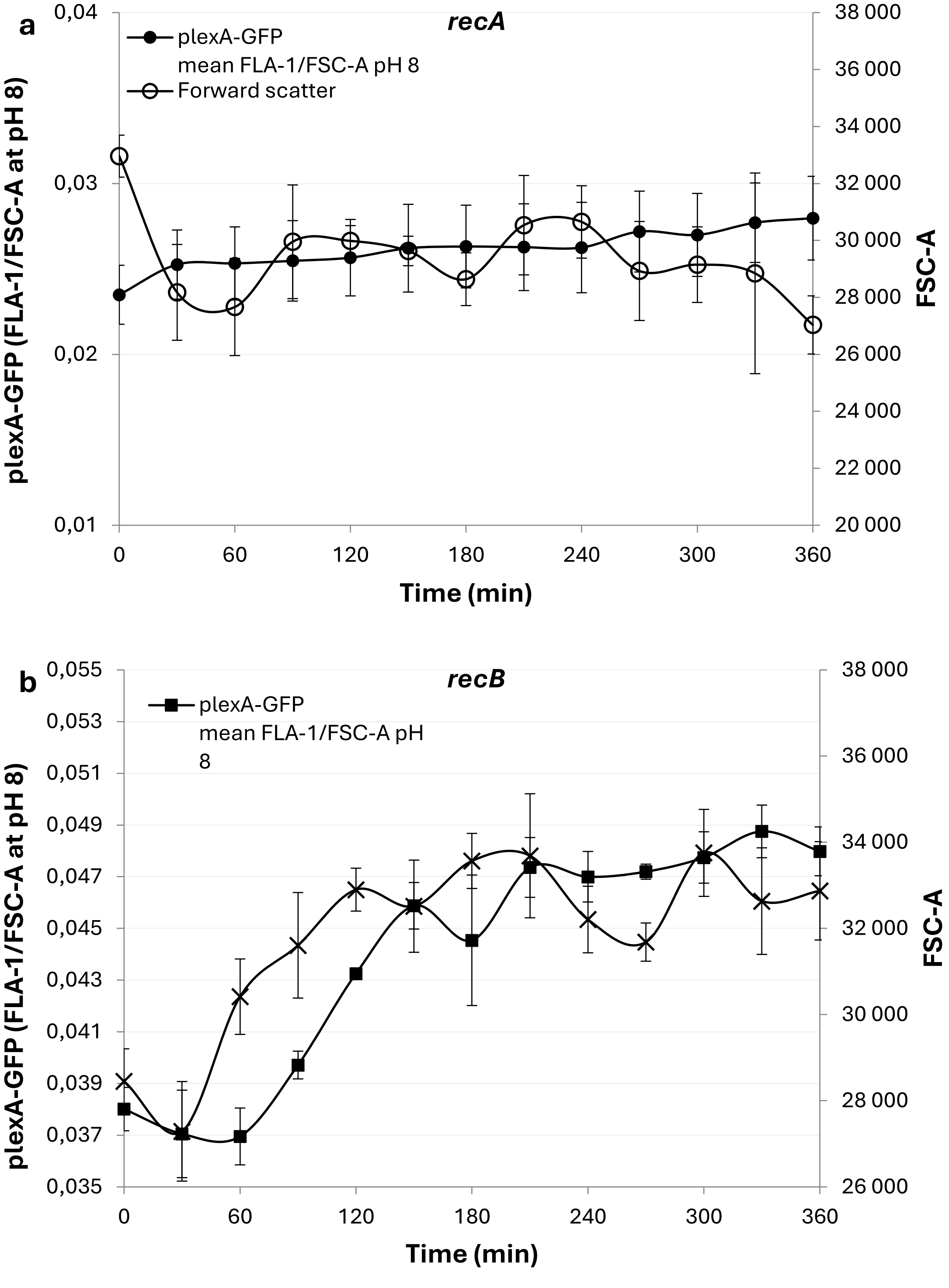

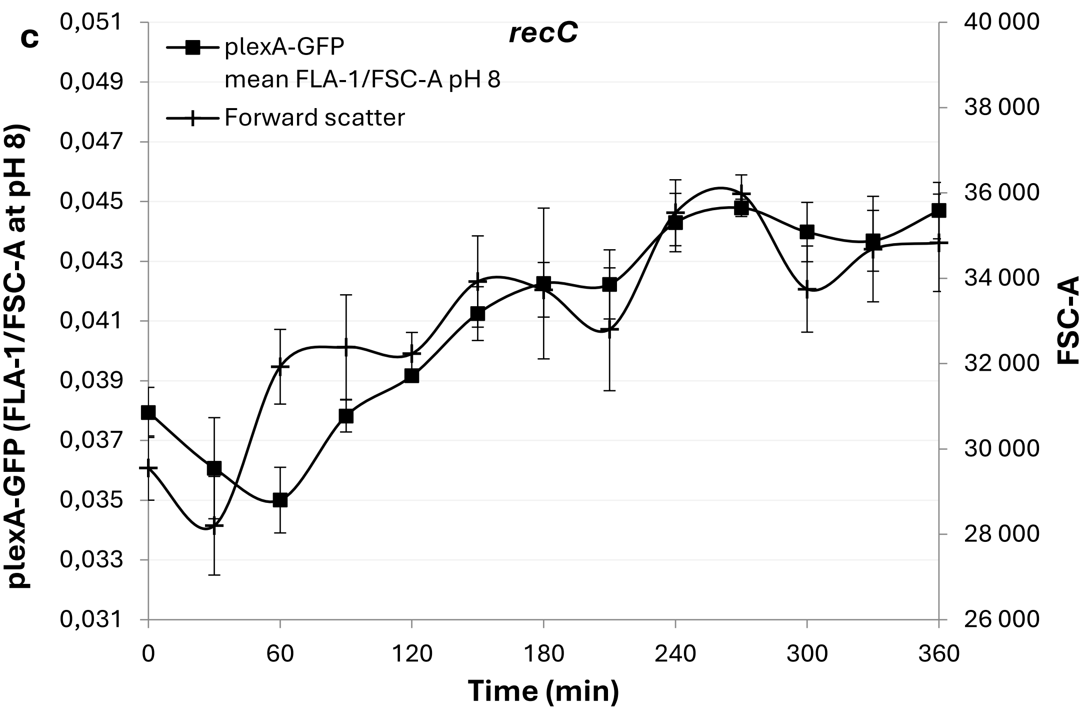
c
recC
plexA-GFP
mean FLA-1/FSC-A pH 8
Forward scatter
plexA-GFP (FLA-1/FSC-A at pH 8)
FSC-A
Time (min)
0,051
0,049
0,047
0,045
0,043
0,041
0,039
0,037
0,035
0,033
0,031
40 000
38 000
36 000
34 000
32 000
30 000
28 000
26 000
0
60
120
180
240
300
360

Flow cytometry forward scattering measurements (FSC-A) closely track those of plexA-gfp fluorescence density measurements (FLA-1/FSC-A) in key SOS response gene mutants *recA*, *recB* and *recC*. (**a**-**c**) Fluorescence density measurements of Gfp were obtained at pH 8 using sodium benzoate. All data points shown are the means ± standard deviations, *n* = 3 (independent biological replicates).

Fig. S3: Typical microscopy images of the mutants before and after treatment.

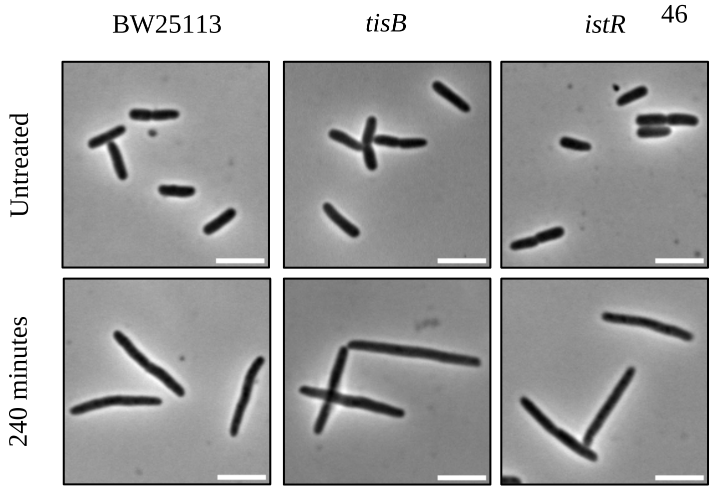

Representative bacterial filamentation phase contrast images from microscopy immediately before the addition of the nalidixic acid and 240 minutes after nalidixic acid treatment. Scale bar = 5 µm.

51 **Fig. S4: Typical microscopy images of the mutants before and after treatment.**

52

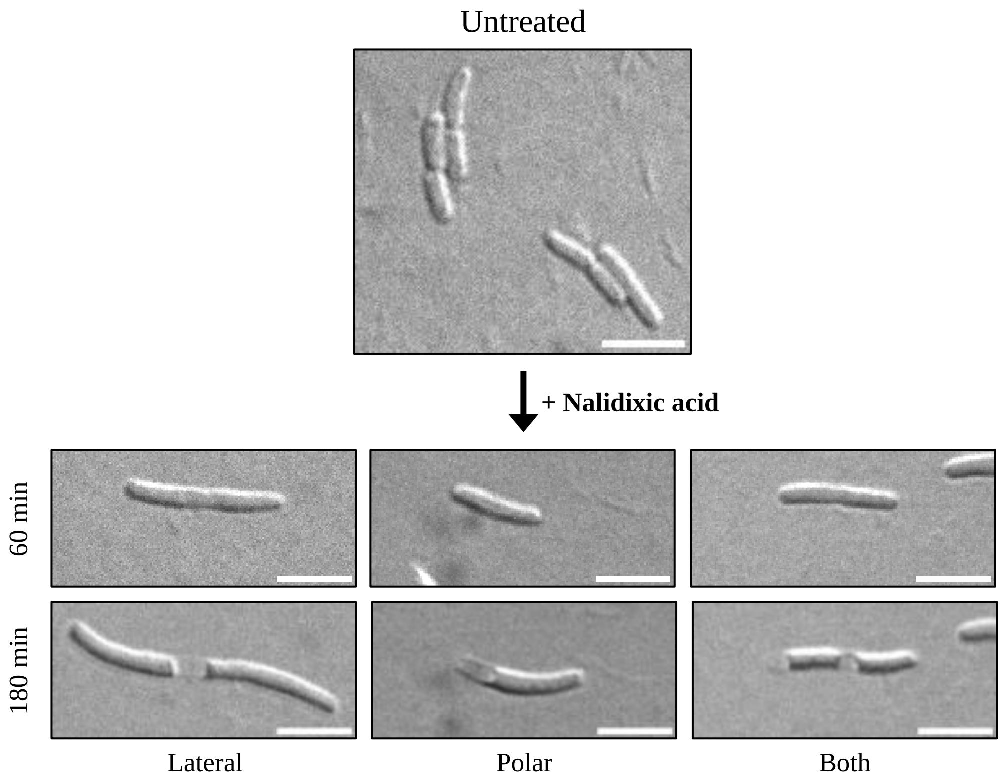

DIC images of WT cells from one experiment at pH 5 before (untreated) and after treatment at indicated timepoints. Both polar and lateral cytoplasmic condensation were observed after addition of nalidixic acid. Scale bar = 5 µm.
